# Temporospatial variation in environmental risk factors and related gastric cancer incidence: a registry-based study in an area with the largest gastric cancer burden in China

**DOI:** 10.7189/jogh.15.04083

**Published:** 2025-06-02

**Authors:** Lulu Zhao, Huang Huang, Chun Zhang, Xiaoyi Luan, Penhui Niu, Yitong Zhu, Yuyang Xiong, Wanqing Wang, Xue Han, Danqi Huang, Huilin Wang, Peiyuan Sun, Zhuolun Hu, Ranran Qie, Yuting Xie, Mengyao Wu, Qi Yan, Tianjie Zhao, Yuqin Liu, Jiang Li, Yawei Zhang, Yingtai Chen

**Affiliations:** 1 Department of Pancreatic and Gastric Surgery, National Cancer Center/National Clinical Research Center for Cancer/Cancer Hospital, Chinese Academy of Medical Sciences and Peking Union Medical College, Beijing, China; 2 Department of Cancer Prevention and Control, National Cancer Center/National Clinical Research Center for Cancer/Cancer Hospital, Chinese Academy of Medical Sciences and Peking Union Medical College, Beijing, China; 3 Key Laboratory of Western China’s Environmental System, College of Earth and Environmental Science, Lanzhou University, Lanzhou, China; 4 Global Health Department, Chinese Academy of Medical Scineces &Peking Union Medical College, School of Population Medicine and Public Health, Beijing, China; 5 College of Hydrology and Water Resources, Hohai University, Nanjing, China; 6 Office of Cancer Screening, National Cancer Center/National Clinical Research Center for Cancer/Cancer Hospital, Chinese Academy of Medical Sciences and Peking Union Medical College, Beijing, China; 7 Aerospace Information Research Institute, Chinese Academy of Sciences, Beijing, China; 8 Cancer Epidemiology Research Center, Sun Yat-sen University Cancer Center Gansu Hospital, Gansu Provincial Cancer Hospital, Lanzhou, China

## Abstract

**Background:**

Gastric cancer is a formidable global heath challenge, especially in China. We aimed to investigate temporal trends and spatial variations of gastric cancer incidence and mortality in Gansu Province, which has the highest incidence rate of gastric cancer in China, and sought to explore the role of various geographic and environmental factors played in the variations of incidence rates of gastric cancer.

**Methods:**

We sourced the incidence and mortality data for gastric cancer from the China Cancer Registry Annual Report and environmental data from National Soil Series Survey and Compilation of Soil Series of China and China National Environmental Monitoring Centre. We used joinpoint regression models to examine the temporal trends in age-standardised incidence rate (ASIR) and age-standardised mortality rate (ASMR) for gastric cancer between 2010 and 2018. We used the Pearson correlation coefficient, robust linear regression, and least-squares regression models to explore the associations between environmental parameters and gastric cancer incidence.

**Results:**

The ASIR of gastric cancer per 100 000 population in Gansu Province showed a slightly increasing trend from 2010 (77.75; 95% confidence interval (CI) = 73.98, 81.52) to 2014 (85.20; 95% CI = 80.36, 90.04) and then started to decrease to 51.77 (95% CI = 49.14, 54.40) in 2018. The mortality rate of gastric cancer per 100 000 population remained stable from 2010 (55.69; 95% CI = 52.38, 59.01) to 2017 (51.06; 95% CI = 48.11, 54.01), while the rate showed a noticeable decline in 2018 (33.37; 95% CI = 31.26, 35.48). In 2018, significant variations in the ASIR (more than 5-fold) and ASMR (more than 11-fold) were observed across 15 registries in Gansu Province. Associations between soil properties at different depths and ASIR were calculated for 15 registries in Gansu Province, and statistically significant indicators included soil pH, total nitrogen density (TND), total phosphorus (TP), and soil organic carbon density (SOCD) (*P* < 0.05). No statistically significant correlation was found between ASIR and ambient air pollutants and surface water properties. The environmental variables explained about 37.9% of the variability in regional level ASIR of gastric cancer (adjusted R^2^ = 0.379, F = 3.136, *P* = 0.046). In addition, strong correlations among soil pH, TND, TP, and SOCD. While chemical fertilisers have been documented to induce soil acidification and elevate N, P, and C levels, we thus compared the annual trends in the application of chemical fertilisers with the ASIR of gastric cancer in Gansu Province, revealing consistent patterns. The correlation coefficient between annual quantity of applied chemical fertilisers and ASIR was 0.638.

**Conclusions:**

We observed significant geospatial variations in the ASIR and ASMR of gastric cancer in Gansu Province, which has the highest incidence rate of gastric cancer in China, as well as a novel correlation between the usage of chemical fertilisers and the incidence of gastric cancer, highlighting the need for further research in this area.

Despite a decline in the incidence and mortality rates of gastric cancer attributed to lifestyle interventions and extensive initiatives for *H. pylori* screening and treatment, gastric cancer remains a significant contributor to the global cancer burden [1–3]. In 2020, there were over one million estimated new cases of the diseases and approximately 770 000 related deaths globally, making it fifth most common newly diagnosed cancer and the fourth most common cause of cancer mortality worldwide, with China alone accounting for approximately 50% of global new cases [4].

One of the most notable areas in this sense is Gansu Province; located in the northwest region of China, the area has the highest incidence and mortality rates of gastric cancer within the country [5]. A previous study based on hospital data from this province reported the crude incidence rates of gastric cancer per 100 000 population to be 132.28 in Zhangye City, 126.76 in Wuwei City, 33.33 in Jiayuguan City and 29.23 in Longnan City. The huge spatial variation in the incidence rate of gastric cancer in Gansu Province highlighted an urgent need to understand potential contributors to the regional variations of gastric cancer incidence in Gansu Province.

Lifestyle and natural environmental factors are widely recognised as closely related to the development of cancer. Substantial scientific evidence supports that *H. pylori* infection, smoking, alcohol consumption, and high-salt foods are associated with an increased risk of gastric cancer [6–11]. Other research has suggested a potential link between gastric cancer risk and environmental factors, including air pollution [12,13], soil properties [14], and surface water quality [15]. Two cohort studies examined the relationship between air pollution and gastric cancer incidence [12,13]. Both studies investigated PM_2.5_, with one suggesting a positive association and another finding no association. Variations in PM compositions across different study locations might be a potential reason for the inconsistent results. Only one published study reported that counties with calcareous soils in the western USA and those with acidic sandy soils in northern Wisconsin exhibited a higher incidence of gastric cancer [14]. To our knowledge, no study has explored other soil properties (*i.e.* soil nitrogen, phosphorus, and potassium) in relation to gastric cancer.

In addition, Gansu Province has several water systems: the water systems in high-incidence areas of gastric cancer like the Wuwei and Zhangye regions get their water from the melting ice and snow of the Qilian Mountains, while the low-incidence area of Longnan region is situated in the Yangtze River Basin. Therefore, exploring whether the composition and quality of surface water affect the incidence of gastric cancer might provide new insights in the etiology of gastric cancer. A previous study found a positive relationship between a number of pollutants present at high levels in surface water and gastric cancer incidence in China [15]; however, many physical and chemical properties of surface water were not investigated.

## METHODS

### Data sources

We obtained data on the incidence and mortality of gastric cancer used from the China Cancer Registry Annual Reports, which include said databased on five registries (Baiyin, Pingchuan, Jingyuan, Huining, and Jingtai) in 2010–17 and 15 registries (Baiyin, Pingchuan, Jingyuan, Huining, Jingtai, Liangzhou, Minqin, Gulang, Tianzhu, Ganzhou, Gaotai, Jingning, Dunhuang, Qingcheng and Lintan) in 2018, stratified by age, gender, and cancer site of. In the Gansu Cancer Registry Annual Report of 2018, the population coverage amounted to 5 372 850 individuals, with 3133 gastric cancer incident cases and 1996 associated deaths. We computed age-standardised incidence rates (ASIRs) and age-standardised mortality rates (ASMRs) for gastric cancer using the 2000 global and China-specific standard populations as references, respectively.

We retrieved data on soil physical and chemical properties of Gansu Province from the National Soil Series Survey and Compilation of China Soil Series Monograph 2010–18 [16], including pH, total nitrogen (TN), total nitrogen density (TND), total phosphorus content (TP), total phosphorus density (TPD), total potassium (TK), total potassium density (TKD), cation exchange capacity (CEC), soil organic carbon (SOC), soil organic carbon density (SOCD), and bulk density. Horizontal spatial resolution was 90 m, in addition to resampling of 250- and 1000-m resolution, respectively; vertical direction included six soil depths: 0–5, 5–15, 15–30, 30–60, 60–100, 100–200 cm.

We sourced the air quality data for Gansu Province for the 2015–18 period from the China National Environmental Monitoring Centre, which provided comprehensive coverage across all major administrative regions at the prefecture level in China. The data encompassed key pollutants of ambient air, including PM_2.5_, PM_10_, NO_2_, SO_2_, and O_3_. The physical and chemical properties of surface water in Gansu Province was also obtained through the China National Environmental Monitoring Centre, including water pH, conductivity, dissolved oxygen, turbidity, permanganate index, total nitrogen and total phosphorus.

Lastly, we derived the quantity of chemical fertiliser applied in Gansu Province from the report on agricultural fertiliser application data in Gansu Province, which was a publicly accessible government data. Fertiliser usage was assessed due to its potential impact on soil acidification and nutrient composition, which could influence environmental risk factors for gastric cancer.

### Statistical analysis

We used joinpoint regression models to examine the time trends in ASIR and ASMR for gastric cancer during the period 2010 to 2018 [17]. This was done using the Joinpoint software, version 4.8.0.1 (National Cancer Institute, Rockville, MD, USA) to systematically assess time trends in a structured manner and statistically evaluate the significance of trends between join points. Specifically, we applied a maximum of two-line segments (one join point) our models. To indicate the direction and magnitude of the trends, we calculated the annual percent change (APC) and average APC (AAPC). To ensure comparability of incidence and mortality rates, we only included data from five registries (Baiyin, Pingchuan, Jingyuan, Huining, and Jingtai) with complete data for the entire study period (2010–18) in our time trend analyses.

We computed the standard error (SE) of age-standardised rates (ASRs) using the following formula [18]:



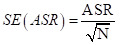



Here, the ASR is the rate of gastric cancer incidence or mortality adjusted for the age distribution of a specific reference population. N is the number of gastric cancer cases (for incidence) or deaths (for mortality) observed in the population under study. The SE provides a measure of the variability or precision of the ASR estimate, which is important for assessing the statistical significance of observed trends or differences in rates.

The 95% confidence interval (CI) for the ASR could be calculated as:

*ASR* ± 1.96 × SE(ASR)

To compare gastric cancer incidence and mortality between Gansu Province and entire China, as well as between genders, we cinozted IRRs and MRRs using buffer analysis. We obtained the 95% CIs for the incidence rate ratios (IRRs) and mortality rate ratios (MRRs) with the following formula [19]:

(*ASR*_1_ / *ASR*_2_)^1 ± 1.96/^*^X^*

where



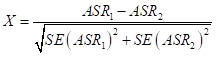



We used the ArcMap, version 10.8 (Jack Dangermond & Laura Dangermond, Redlands, CA, USA) to visually represent the relationship between wells with soil physical and chemical properties and to analyse their spatial correlation. We utilised Pearson correlation coefficients and robust linear regression to compare gastric cancer incidence rates among different environmental factors across geographical regions (n = 15). We otherwise conducted a separate robust linear regression for each environmental factor at varying soil depths (*e.g.* 0–5 cm, 5–15 cm, *etc.*). The model structure is as follows:

*ASIRi* = β0 + β1X*i* + *εi*

*ASIRi* is age-standardised gastric cancer incidence rate for region *i*, X*i* is environmental factor (*e.g.* soil pH, TND, TP, or SOCD) at a specific soil depth for region i, β0 is intercept of the model, βi is coefficient representing the relationship between ASIR and the environmental factor, andεi is residual error term.

We determined the overall strength of the association by a generalised least-squares regression model weighted by regional population through calculating the overall R^2^ of the model [20].

We conducted all statistical analyses using *R*, version 4.1.0 (R Core Team, Vienna, Austria), and defined statistical significance as a *P*-value <0.05.

## RESULTS

There were 15 683 and 805 055 gastric cancer incident cases recorded in Gansu Province and China in 2010–18, respectively (Table S1 in the **Online Supplementary Document**), with 10 149 and 585 430 deaths, respectively, over the same period. The ASIR of gastric cancer in Gansu Province significantly exceeded that of the entire China during the study period (Figure S1 in the **Online Supplementary Document**), with IRRs ranging from 3.06 (95% CI = 2.91, 3.20) to 3.89 (95% CI = 3.72, 4.07). We observed a significantly higher mortality in Gansu Province, with MRRs from 2.59 (95% CI = 2.43, 2.75) to 3.86 (95% CI = 3.64, 4.07).

**Figure 1:**
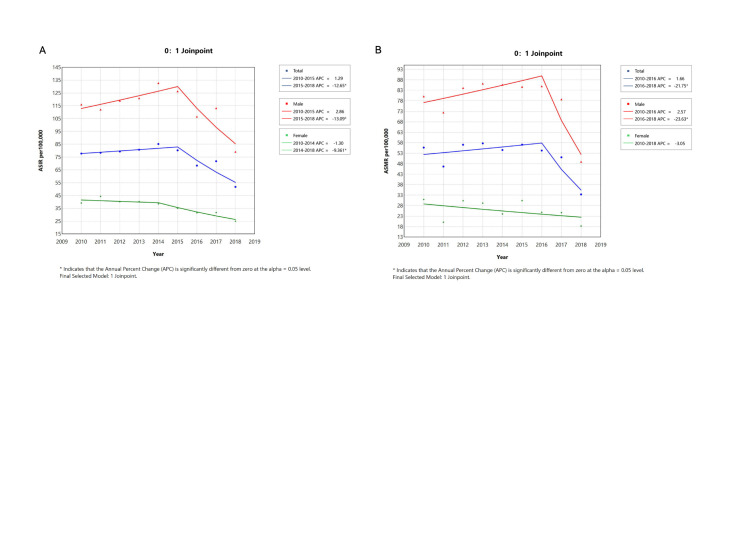
Trends in ASIR and ASMR world per 100 000 population of gastric cancer by gender in Gansu Province, 2010–18. **Panel A**. Trends in ASIR. **Panel B.** Trends in ASMR. ASIR – age-standardised incidence rate, ASMR – age-standardised mortality rate.

The ASIR of gastric cancer per 100 000 population in Gansu Province showed a slightly increasing trend from 2010 (77.75; 95% CI = 73.98, 81.52) to 2014 (85.20; 95% CI = 80.36, 90.04) and then started to decrease with ASIR of 51.77 (95% CI = 49.14, 54.40) in 2018 (**Figure 1**, Panel A, **Table 1**, **Table 2**). The observed trend of ASIR was likewise found among men, while it decreased among women through the entire period (AAPC = −5.55, *P* = 0.001), with some fluctuations. The ASIR was much higher in men than in women overall, with IRRs ranging from 2.53 (95% CI = 2.27, 2.79) to 3.60 (95% CI = 3.21, 3.99). After excluding data from 2018 (Table S2 in the **Online Supplementary Document**), the ASIR of gastric cancer in Gansu Province during 2014–17 showed a significant decline (APC = −6.12 in overall population, −5.40 in men, and −6.13 in women; all *P* < 0.05).

**Table 1:** ASIR per 100 000 population for gastric cancer in Gansu Province, 2010–18

		ASIR (95% CI)			
**Year**	**Number of incidence cases**	**Total**	**Male**	**Female**	**IRR**
2010	1633	77.75 (73.98, 81.52)	116.04 (109.53, 122.55)	39.19 (35.41, 42.97)	2.96 (2.63, 3.29)
2011	1763	78.34 (73.47, 83.22)	112.10 (105.92, 118.28)	44.33 (40.43, 48.23)	2.53 (2.27, 2.79)
2012	1655	79.35 (74.64, 84.06)	119.01 (112.41, 125.60)	40.20 (36.28, 44.12)	2.96 (2.63, 3.29)
2013	1861	80.76 (76.24, 85.28)	120.91 (114.55, 127.27)	40.24 (36.62, 43.87)	3.00 (2.69, 3.32)
2014	1921	85.20 (80.36, 90.04)	132.76 (126.01, 139.51)	38.54 (34.91, 42.16)	3.45 (3.08, 3.81)
2015	1942	80.27 (75.71, 84.83)	126.32 (119.96, 132.68)	35.09 (31.76, 38.42)	3.60 (3.21, 3.99)
2016	1660	68.36 (64.60, 72.12)	106.55 (100.68, 112.41)	31.53 (28.40, 34.65)	3.38 (3.00, 3.76)
2017	1764	71.77 (67.63, 75.92)	113.17 (107.18, 119.17)	31.70 (28.57, 34.82)	3.57 (3.17, 3.90)
2018	1484	51.77 (49.14, 54.40)	79.20 (74.55, 83.85)	25.02 (22.46, 27.58)	3.17 (2.79, 3.54)
AAPC		−3.59 (−6.45, −0.64)	−2.76 (−6.04, 0.65)	−5.55 (−7.56, −3.49)	
*P*-value		0.049	0.155	0.001	

ASIR – age-standardised incidence rate, AAPC – average annual percent change, APC – annual percent change, IRR – incidence rate ratios, MRR – mortality rate ratio

**Table 2:** ASMR per 100 000 population for gastric cancer in Gansu Province, 2010–18

		ASMR (95% CI)			
**Year**	**Number of deaths**	**Total**	**Male**	**Female**	**MRR (95% CI**
2010	1084	55.69 (52.38, 59.01)	80.13 (74.53, 85.72)	30.93 (27.41, 34.45)	2.59 (2.24, 2.94)
2011	991	46.66 (43.75, 49.56)	72.44 (67.34, 77.55)	20.11 (17.44, 22.79)	3.60 (3.06, 4.14)
2012	1091	57.06 (53.68, 60.45)	84.14 (78.34, 89.93)	30.34 (26.79, 33.89)	2.77 (2.40, 3.15)
2013	1225	57.72 (54.49, 60.96)	86.22 (80.61, 91.82)	29.21 (25.99, 32.43)	2.95 (2.57, 3.33)
2014	1191	54.54 (51.44, 57.64)	85.69 (80.17, 91.20)	24.04 (21.13, 26.94)	3.56 (3.08, 4.05)
2015	1189	57.09 (53.84, 60.33)	84.68 (79.05, 90.31)	30.39 (27.06, 33.72)	2.79 (2.43, 3.14)
2016	1267	54.29 (51.30, 57.28)	84.96 (79.62, 90.31)	24.82 (21.99, 27.65)	3.42 (2.98, 3.87)
2017	1152	51.06 (48.11, 54.01)	78.72 (73.49, 83.95)	24.64 (21.76, 27.51)	3.20 (2.77, 3.62)
2018	959	33.37 (31.26, 35.48)	48.95 (45.31, 52.59)	18.27 (16.07, 20.47)	2.68 (2.30, 3.06)
AAPC		−3.10 (−7.00, 0.97)	−2.83 (−7.02, 1.55)	−3.05 (−7.42, 1.52)	
*P*-value		0.177	0.243	0.229	

AAPC – average annual percent change, APC – annual percent change, ASMR – age-standardised mortality rate, IRR – incidence rate ratios, MRR – mortality rate ratio

The mortality rate of gastric cancer per 100 000 population remained stable from 2010 (55.69; 95% CI = 52.38, 59.01) to 2018 (33.37; 95% CI = 31.26, 35.48), with an AAPC of −3.10 (*P* = 0.177). We observed similar trends for both men and women, with the former further experiencing much higher mortality rates compared to women with MRRs varying from 2.59 (95% CI = 2.24, 2.94) to 3.60 (95% CI = 2.24, 2.94) during the study period.

Although fluctuations of the trends of ASIR and SMR were more visible in each registry in Gansu Province in 2010–18 (Figure S2 in the **Online Supplementary Document**), a significant declining trend of both ASIR and ASMR was found in Liangzhou (AAPC = −4.39 and −0.83, respectively).

In 2018, we observed significant variations in the ASIR (more than 5-fold) and ASMR (more than 11-fold) across 15 registries in Gansu Province (**Table 3**). Lintan and Ganzhou had the highest ASIR (88.91; 95% CI = 71.57, 106.25) and ASMR (56.41; 95% CI = 49.15, 63.67) per 100 000 population, while Baiyin had the lowest (ASIR: 16.11; 95% CI = 12.84, 19.38, ASMR: 4.92; 95% CI = 3.19, 6.65). The observed variations of ASIR and ASMR remained the same in men and women. Further, the male-to-female rate ratio was also varied by registries, with IRRs ranging from 1.83 (95% CI = 1.30, 2.35) to 4.30 (95% CI = 3.41, 5.19) and MRRs ranging from 2.04 (95% CI = 1.30, 2.78) to 4.78 (95% CI = 3.33, 4.78).

**Table 3:** The ASIR and ASMR per 100 000 population for gastric cancer in Gansu Province across 15 registries, 2018

		ASIR (95% CI)			
**Region**	**Number of incidence cases**	**Total**	**Male**	**Female**	**IRR (95% CI)**
Lintan	101	88.91 (71.57, 106.25)	127.54 (98.48, 156.60)	52.33 (32.59, 72.07)	2.44 (1.88, 2.99)
Gulang	258	68.72 (60.33, 77.10)	103.54 (89.04, 118.03)	33.78 (25.37, 42.19)	3.06 (2.64, 3.49)
Ganzhu	370	65.07 (58.44, 71.70)	91.29 (80.44, 102.14)	37.49 (30.07, 44.92)	2.43 (2.15, 2.72)
Liangzhou	889	60.36 (56.39, 64.33)	93.67 (86.60, 100.74)	28.88 (25.01, 32.75)	3.24 (3.00, 3.49)
Gaotai	108	57.63 (46.76, 68.50)	92.05 (72.93, 111.17)	21.40 (11.78, 31.03)	4.30 (3.41, 5.19)
Jingning	309	55.05 (48.91, 61.19)	81.66 (71.06, 92.26)	28.60 (22.37, 34.83)	2.85 (2.48, 3.23)
Tianzhu	117	53.53 (43.83, 63.23)	70.95 (55.40, 86.50)	34.93 (23.67, 46.18)	2.03 (1.59, 2.48)
Huining	334	53.10 (47.40, 58.79)	72.61 (63.29, 81.94)	33.20 (26.72, 39.67)	2.19 (1.91, 2.47)
Minqin	138	40.79 (33.98, 47.59)	63.50 (51.46, 75.53)	17.93 (11.62, 24.25)	3.54 (2.87, 4.21)
Jingtai	81	33.98 (26.58, 41.38)	53.20 (39.96, 66.44)	15.50 (8.53, 22.48)	3.43 (2.58, 4.29)
Dunhuang	43	29.77 (20.87, 38.67)	45.07 (29.69, 60.45)	13.35 (5.08, 21.63)	3.38 (2.22, 4.53)
Jingyuan	148	29.07 (24.38, 33.75)	39.93 (32.02, 47.83)	18.68 (13.50, 23.86)	2.14 (1.41, 2.87)
Pingchuan	75	28.03 (21.69, 34.38)	41.78 (30.93, 52.62)	14.02 (7.54, 20.50)	2.98 (1.40, 4.56)
Qingcheng	69	24.42 (18.65, 30.18)	31.46 (22.37, 40.55)	17.22 (10.18, 24.26)	1.83 (1.30, 2.35)
Baiyin	93	16.11 (12.84, 19.38)	21.95 (16.69, 27.20)	9.79 (6.03, 13.55)	2.24 (1.23, 3.26)
		**ASMR (95% CI)**			
	**Number of deaths**	**Total**	**Male**	**Female**	**IRR**
Ganzhu	232	56.41 (49.15, 63.67)	81.63 (69.02, 94.24)	30.09 (23.09, 37.09)	2.71 (2.29, 3.13)
Lintan	67	55.81 (42.45, 69.17)	83.95 (61.13, 106.77)	31.20 (15.41, 47.00)	2.69 (1.96, 3.42)
Gulang	189	52.96 (45.41, 60.51)	79.79 (66.76, 92.83)	26.15 (18.51, 33.79)	3.05 (2.55, 3.55)
Huining	246	39.85 (34.87, 44.83)	57.27 (48.91, 65.64)	21.59 (16.38, 26.80)	2.65 (2.27, 3.04)
Jingtai	81	39.11 (30.59, 47.63)	60.63 (45.54, 75.72)	18.77 (10.33, 27.21)	3.23 (2.43, 4.03)
Liangzhou	544	37.62 (34.46, 40.78)	55.25 (49.77, 60.73)	21.08 (17.75, 24.41)	2.62 (2.36, 2.88)
Jingning	191	34.13 (29.29, 38.98)	52.52 (44.00, 61.04)	15.64 (11.07, 20.21)	3.36 (2.81, 3.90)
Tianzhu	68	31.85 (24.28, 39.41)	48.22 (35.24, 61.20)	14.37 (7.10, 21.64)	3.36 (2.45, 4.26)
Dunhuang	35	26.30 (17.59, 35.01)	42.12 (26.79, 57.45)	9.49 (1.90, 17.08)	4.44 (2.82, 6.06)
Gaotai	51	26.23 (19.03, 33.43)	42.66 (29.76, 55.56)	8.93 (3.10, 14.76)	4.78 (3.33, 6.22)
Minqin	76	21.42 (16.60, 26.23)	35.02 (26.16, 43.88)	8.50 (4.33, 12.66)	4.12 (3.08, 5.16)
Jingyuan	101	20.38 (16.41, 24.36)	31.35 (24.26, 38.45)	10.15 (6.25, 14.06)	3.09 (1.71, 4.46)
Pingchuan	43	16.30 (11.43, 21.18)	23.07 (14.95, 31.19)	9.30 (4.04, 14.56)	2.48 (0.83, 4.13)
Qingcheng	41	14.50 (10.06, 18.94)	19.42 (12.35, 26.48)	9.53 (4.14, 14.92)	2.04 (1.30, 2.78)
Baiyin	31	4.92 (3.19, 6.65)	7.14 (4.16, 10.13)	2.49 (0.86, 4.12)	2.87 (0.64, 5.09)

ASIR – age-standardised incidence rate, ASMR – age-standardised mortality rate, IRR – incidence rate ratio, MRR – mortality rate ratio

The mean ages of gastric cancer diagnosis and death increased from 60.19 and 62.95 years in 2010 to 63.89 and 65.73 years in 2018, respectively (Figures S3, Panels A and B, Table S3 in the **Online Supplementary Document**). Men and women showed the same pattern. We also observed greater than six-year differences in mean ages of diagnosis (61.15-year in Lintan and 67.62 in Baiyin) and death (62.54-year in Lintan and 69.27 in Qingcheng) across 15 registries in the Gansu Province in 2018 (Table S4 in the **Online Supplementary Document**). The negative correlations between mean age of diagnosis cases and ASIR (correlation coefficient = −0.641, *P* = 0.010) and between average age of deaths and ASMR (correlation coefficient = −0.757, *P* = 0.001) were found (Figures S3, Panels C and D in the **Online Supplementary Document**).

**Figure 2:**
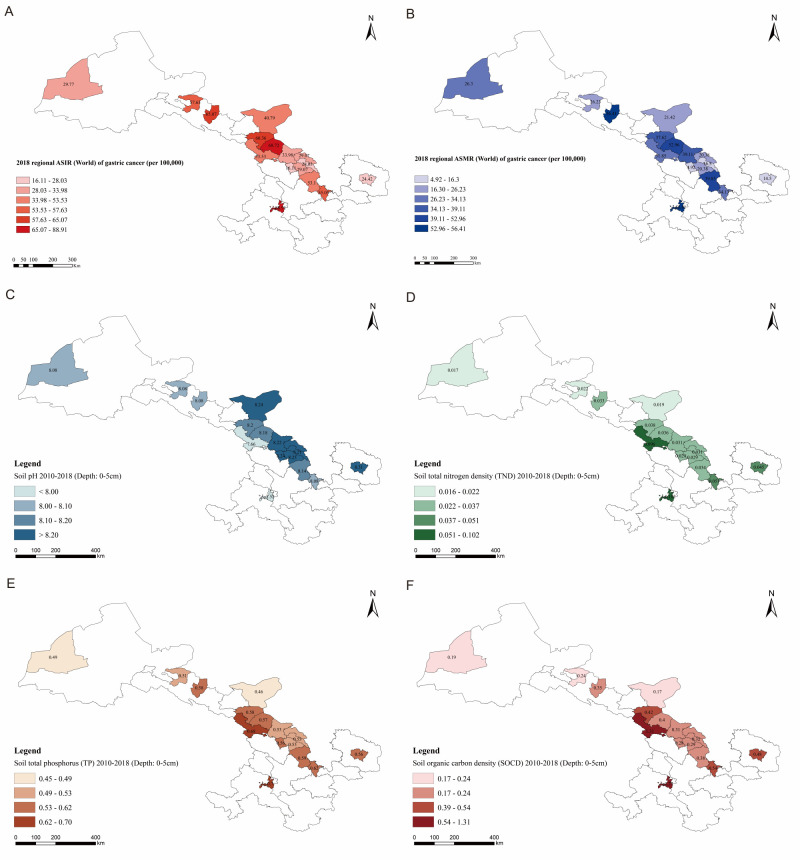
Mapping ASIR (**Panel A**) and ASMR (**Panel B**) world of gastric cancer per 100 000 population in 2018, and soil information as follows (2010–18): surface soil pH (**Panel C**), TND (**Panel D**), TP (**Panel E**) and SOCD (**Panel F**) in of Gansu Province. ASIR – age-standardised incidence rate, ASMR – age-standardised mortality rate, TND – total nitrogen density, TP – total phosphorus, SOCD – soil organic carbon density.

**Figure 3:**
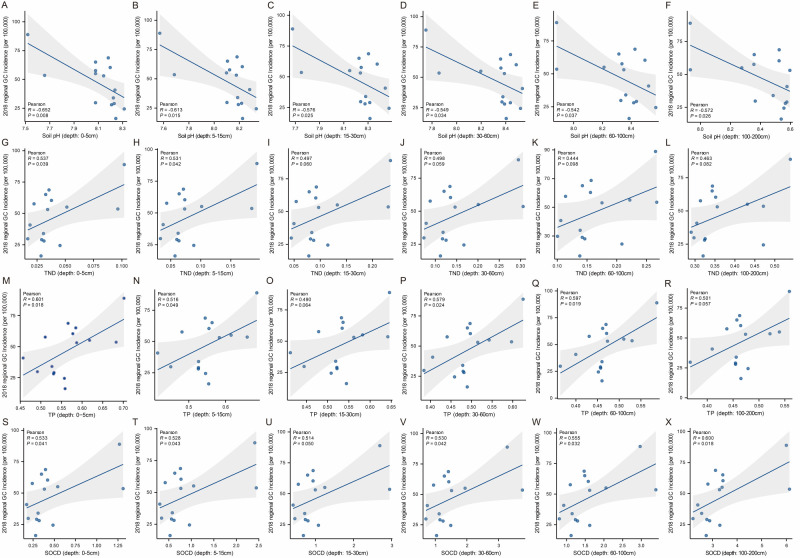
Scatter plots showing relationships between air (**Panels A–E**) and nature water parameters (**Panels F–M**) and ASIR (per 100 000 population) of gastric cancer in Gansu Province. ASIR – age-standardised incidence rate.

The levels of soil properties in Gansu Province were comparable to the national levels with ratios between 0.98 and 1.22 (Table S5 in the **Online Supplementary Document**). Specifically, soil levels of PH, TND, TP, and SOCD showed spatial variations regardless of the depths of sampling (**Figure 2**; Figures S4–7 in the **Online Supplementary Document**). An increasing ASIR was significantly correlated with lower soil pH levels at different depths (**Figure 3**), with correlation coefficients ranging from −0.652 to −0.542 (*P-*values of 0.008, 0.015, 0.025, 0.034, 0.037, and 0.026). We found significant positive correlations between ASIR and levels of soil TND (correlation coefficients of 0.537 for depth 0–5 cm, and 0.531 for 5–15 cm), TP (correlation coefficients of 0.601 for depth 0–5 cm, 0.516 for depth 0–5cm, 0.579 for depth 30–60cm, and 0.597 for depth 60–100 cm), and SOCD (correlation coefficients of 0.533 for depth 0–5cm, 0.528 for depth 5–15 cm, 0.514 for depth 15–30 cm, 0.530 for depth 30–60 cm, 0.555 for depth 60–100 cm, and 0.600 for depth 100–200 cm) levels. We observed no statistically significant correlations for other soil properties, including TK, TKD, TN, SPD, CEC, SOC, and bulk density (Figure S8 in the **Online Supplementary Document**). When the regression model included the soil PH, TND, TP, and SOCD levels, these variables explained about 37.9% of the variability in regional-level ASIR of gastric cancer (adjusted R^2^ = 0.379, F = 3.136, *P* = 0.046).

There were no statistically significant correlations of ASIR with the air pollution levels of PM_2.5_ (correlation coefficient = 0.256, *P* = 0.624), PM10 (correlation coefficient = −0.499, *P* = 0.314), NO_2_ (correlation coefficient = −0.293, *P* = 0.573), SO_2_ (correlation coefficient = −0.451, *P* = 0.370), and O_3_ (correlation coefficient = 0.564, *P* = 0.244) (Figure S9, Panels A–E in the **Online Supplementary Document**). Furthermore, there were no statistically significant correlations for water properties either (Figure S9, Panels F–M in the **Online Supplementary Document**), including water pH (correlation coefficient = −0.387, *P* = 0.269), conductivity (correlation coefficient = −0.605, *P* = 0.064), dissolved oxygen (correlation coefficient = 0.115, *P* = 0.753), turbidity (correlation coefficient = −0.290, *P* = 0.416), permanganate index (R = −0.296, *P* = 0.406), ammonia nitrogen (correlation coefficient = −0.355, *P* = 0.315), total nitrogen (correlation coefficient = −0.366, *P* = 0.299) and total phosphorus (correlation coefficient = −0.269, *P* = 0.452).

There were strong correlations among soil pH, TND, TP, and SOCD (Figure S10, Panels A–C and Table S6 in the **Online Supplementary Document**). Since chemical fertilisers, including nitrogen and phosphorus fertilisers, crucial for crop growth, have been documented to induce soil acidification and elevate N, P, and C levels [21,22]. We found consistent patterns when we compared the annual trends in the application of chemical fertilisers with the ASIR of gastric cancer in Gansu Province. The correlation coefficient between annual quantity of applied chemical fertilisers and ASIR was 0.638 (*P* = 0.064).

## DISCUSSION

In 2018, the ASIR of gastric cancer in all 15 Gansu registries and the ASMR of gastric cancer in most of the registries (n/N = 14/15) exceeded the national averages in China. Both the incidence and mortality rates in this region, characterised by high ASIR and SMR of gastric cancer, varied significantly among the different registries, suggesting that diverse lifestyle and environmental factors might contribute to these differences.

One of the possible explanations for the high incidence of gastric cancer in Gansu Province might be exposures to unhealthy lifestyle habits [6,23,24]. The regions with high incidence of gastric cancer (Gulang, Ganzhou, Liangzhou, and Gaotai) were located in the Hexi region of Gansu Province, where residents often consume large amounts of pickled vegetables, particularly among rural populations [25]. Consuming pickled foods could elevate gastric cancer risk due to their nitrate and nitrite content, which can generate gastric carcinogens known as N-nitroso compounds [26,27]. Moreover, pickled foods often contain high levels of salt, and excessive salt consumption could facilitate *H. pylori* colonization, thereby amplifying the carcinogenic potential of cagA (+) *H. pylori* strains and promoting *H. pylori*-associated carcinogenesis [28]. Furthermore, the excessive intake of pickled foods could potentially displace the consumption of fresh vegetables, further compounding the risk of gastric cancer. Moreover, the notable prevalence of high-level alcohol consumption in the Hexi region could be another significant contributing factor to the elevated incidence rate of gastric cancer [25], especially as the crucial role of alcohol drinking in gastric cancer risk was also indicated in the China Kadoorie Biobank (CKB) cohort recently [6].

Besides lifestyle factors, various environmental factors might also contribute to the high incidence of gastric cancer in Gansu Province. For example, we observed a positive association between soil acidification and increased levels of TND, TP, and SOCD with the ASIR of gastric cancer, while the levels of TND, TP, and SOCD were strongly correlated with soil pH level. Since the use of chemical fertilisers could lead to soil acidification and increased the N, P, and C levels in the soil [21,22], we compared the annual trends of the quantity of applied chemical fertilisers and the ASIR of gastric cancer in Gansu Province, revealing a similar pattern to the main one. Long-term use of chemical fertilisers can also cause the accumulation of heavy metal elements in soil [29]. Exposure to soil contaminated with heavy metals, especially arsenic, cadmium (Cd), lead, and chromium, was associated with increased risk of gastric cancer [30,31]. Taken together, this evidence suggests that chemical fertiliser usage in Gansu Province might be another potential source for high incidence of gastric cancer. Future studies with individual data on exposure to specific chemical fertilisers are warranted to understand the causal links between specific chemical fertilisers and gastric cancer and provide evidence for regulations of chemical fertiliser usage in Gansu Province. Our findings also add to the evidence that improving the environment could significantly impact human health and long-term development, which warrants more resources for research and intervention to reduce excessive fertiliser application and promote sustainable agricultural practices that minimize soil acidification and nutrient imbalance.

From 2010 to 2018, the ASIR and ASMR of gastric cancer in Gansu Province increased initially, but decreased significantly soon in terms of both incidence and mortality rates. However, the decreasing trends of ASIR and ASMR were largely influenced by data from 2018. Nevertheless, the efforts in gastric cancer prevention and control in recent years should not be overlooked. In 2010, Wuwei County in Gansu Province was included as a screening site for the early diagnosis and treatment programme for gastric cancer [32], which might have increased the awareness among people to eradicate *H. pylori* infection as a preventive measure for gastric cancer. In the 2007–08, the prevalence of *H. pylori* was 72.3% among children and teenagers [33] and 81.8% among adults in the Wuwei City [34]. This figure significantly declined in the 2016–17, with an *H. pylori* infection rate of 35.6% of the total population in the Wuwei City [35]. In our study, the Liangzhou District in Wuwei City experienced a significant decline in gastric cancer incidence from 2010 to 2018. In addition, with the improvement of living standards and the dissemination of medical knowledge, the prevalence of *H. pylori* infection in China has declined significantly, decreasing from over 60% in 1983 to below 50% in 2018 [36]. This might be one of the reasons for the decline in the incidence of gastric cancer in Gansu Province in this period, as well as in China overall. We further note that mortality rates are generally less sensitive to detection bias, including those associated with screening-detected cancers [23]. The observed decline in gastric cancer mortality in Gansu Province may indeed be linked to the improvement in medical conditions, as reflected by the significant increase in the number of hospitals from 378 in 2010 to 623 in 2018 (per government reports).

We observed a significantly negative association between incidence rates of gastric cancer and mean age of diagnosis in Gansu Province, indicating that the mean age of diagnosis tended to be earlier in areas with a high incidence rate compared to those with a low incidence rate. Further, we observed a greater than six-year difference in mean ages of diagnosis (61.15 years in Lintan and 67.62 years in Baiyin) across the 15 registries in the Gansu Province in 2018. Genetic variations in certain populations (*e.g.* prevalence of cancer predisposition genes) might contribute to these geographic differences of gastric cancer [37]. Additionally, the persistent presence of poor environmental factors and unhealthy dietary habits might also accelerate the early diagnosis of gastric cancer in the regions [38].

Our study has several limitations. First, this was an ecological study, so no references could be drawn regarding individual-level patterns of natural environmental risk and gastric cancer. The results should be interpreted with caution, and further investigations involving mechanistic or individual-level studies are essential to refine our understanding. Second, we performed trends analysis of ASIR and ASMR using data spanning only nine years in Gansu Province. Future research should consider incorporating data from longer time periods to provide a more comprehensive depiction of the trends in gastric cancer incidence and mortality rates. Third, we did not account for lifestyle factors, which were known to have a significant impact on the occurrence of gastric cancer. However, our study is the first to suggest a potential correlation between the usage of chemical fertilisers and the incidence of gastric cancer based on ecological research evidence, providing ground for generating new study hypotheses for future research.

## CONCLUSIONS

We observed significant geospatial variations in the ASIR and ASMR of gastric cancer in the Gansu Province, the region with the highest incidence of the disease in China. Importantly, we discovered that, alongside other lifestyle factors, the usage of chemical fertilisers in Gansu Province might contribute to the high incidence of gastric cancer. This finding warrants further investigation to better understand the potential factors driving the high incidence rates in the region.

## Additional material


Online Supplementary Document
