# Supplementary material for: Temporospatial variation in environmental risk factors and related gastric cancer incidence: a registry-based study in an area with the largest gastric cancer burden in China
Source: J Glob Health. 2025 Jun 2;15:04083. doi: 10.7189/jogh.15.04083 (PMC12127779; doi:10.7189/jogh.15.04083)
Supplement: Online Supplementary Document [file jogh-15-04083-s001.zip › jogh-15-04083-s001.pdf]

Supplement to: Zhao L, Huang H, Zhang C, Luan X, Niu P, Zhu Y, Xiong Y, Wang W, Han X, Huang D, Wang H, Sun P, Hu Z, Qie R, Xie Y, Wu M, Yan Q, Zhao T, Liu Y, Li J, Zhang Y, Chen Y. Temporospatial variation in environmental risk factors and related gastric cancer incidence: a registry-based study in an area with the largest gastric cancer burden in China. J Glob Health. 2025;15:04083.

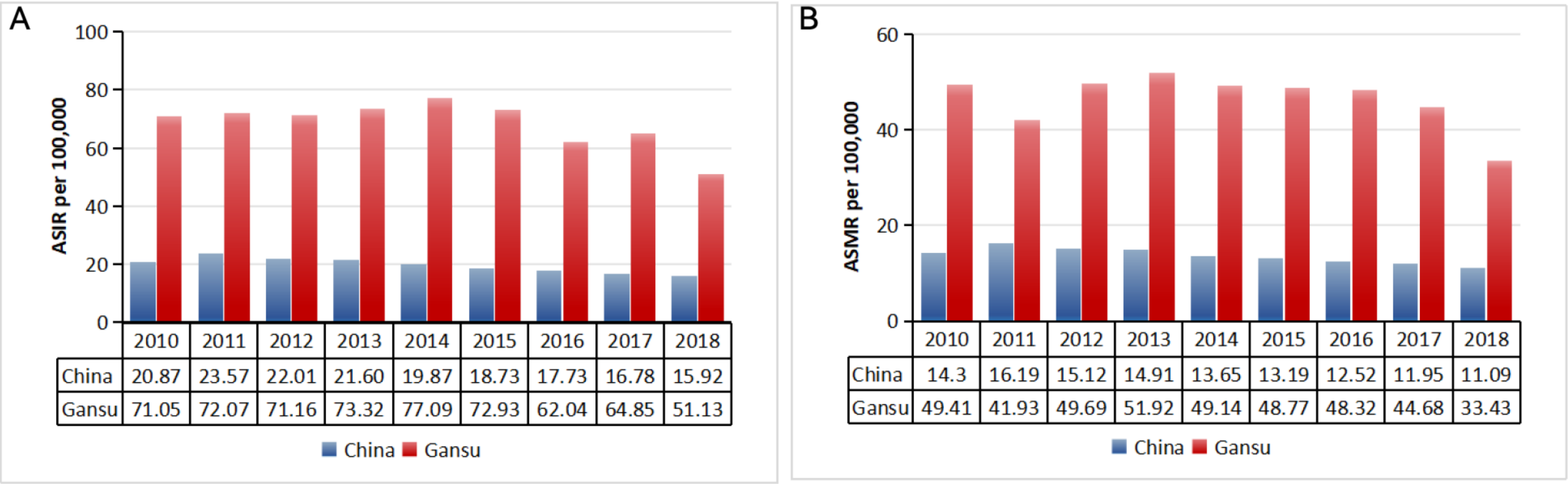

Figure S1. The ASIR (A) and ASMR (B) China of gastric cancer in Gansu Province and total China, 2010-2018. Abbreviations: ASIR, age-standardized incidence rate; ASMR, age-standardized mortality rate.

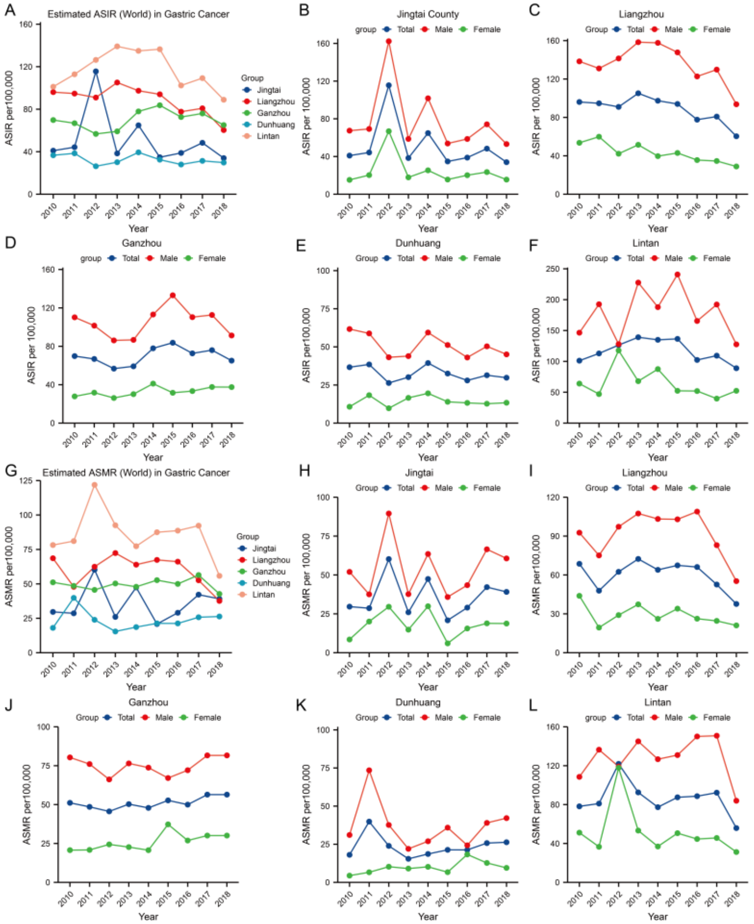

Figure S2. Trends in ASIR (A-F) and ASMR (G-H) world of all gastric cancer by different region and gender in Gansu Province, 2010-2018. Abbreviations: ASIR, age-standardized incidence rate; ASMR, age-standardized mortality rate.

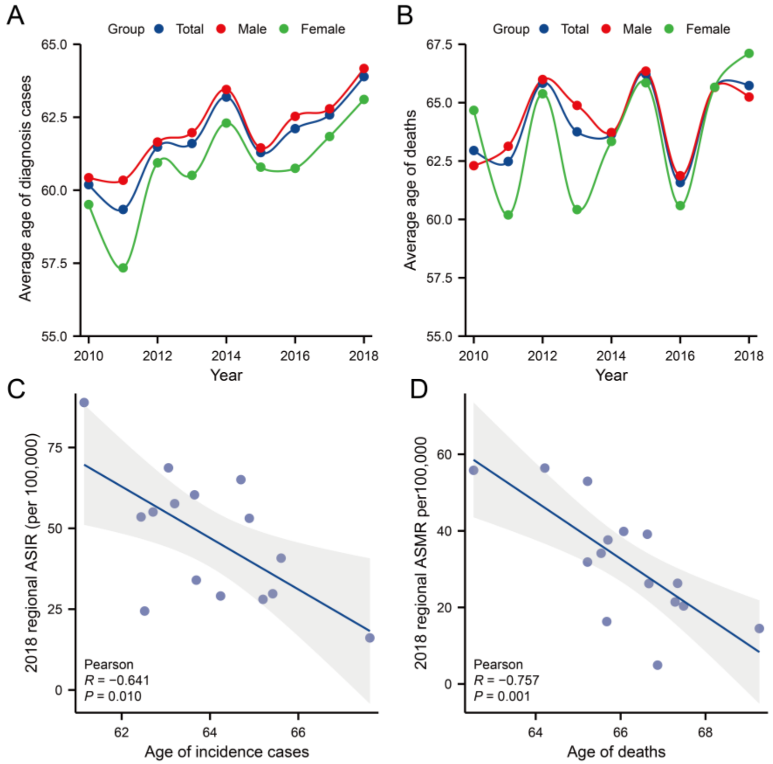

Figure S3. Time trends of average age in incidence cases (Panel A) and deaths (Panel B) during the whole period in Gansu Province, and scatter plots showing relationships between average age in incidence cases (Panel C) or deaths (Panel D) and ASIR or AMSR (per 100 000 population) of gastric cancer in Gansu Province in 2018, respectively. ASIR – age-standardised incidence rate, ASMR – age-standardised mortality rate.

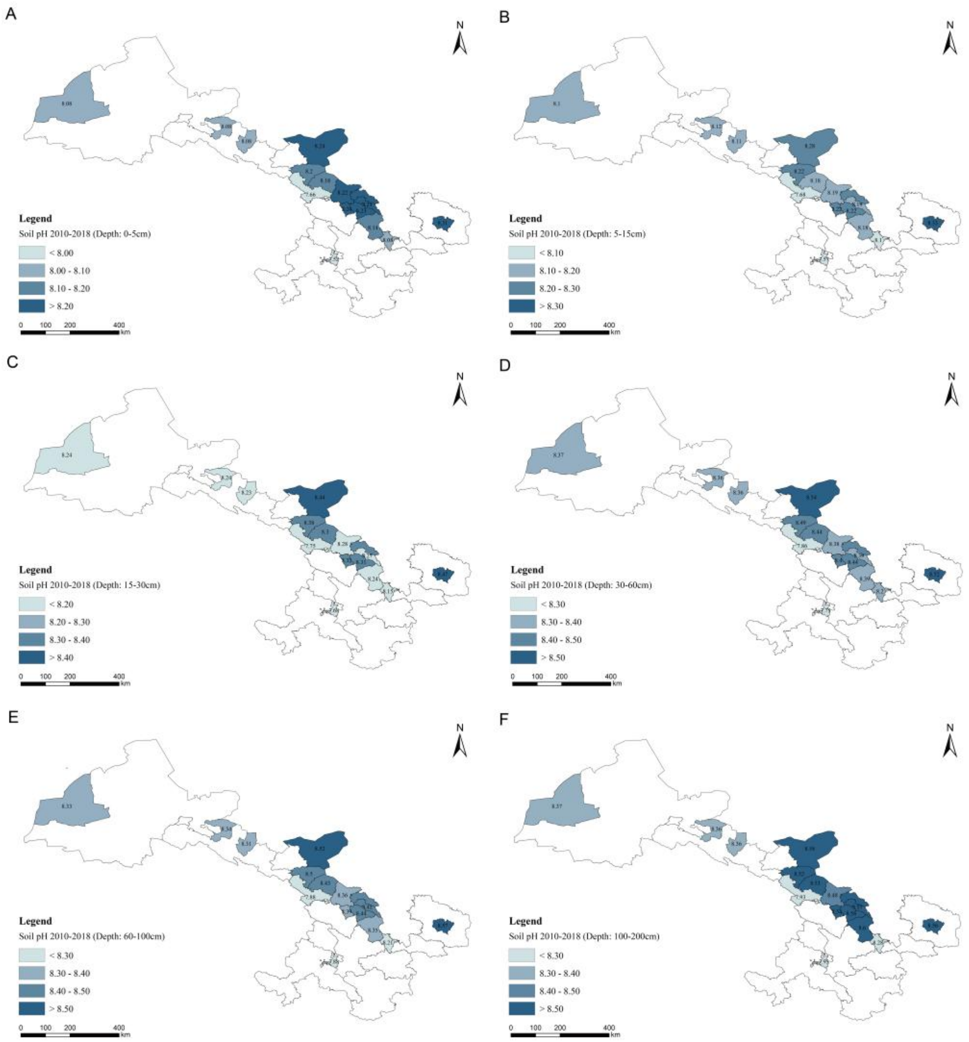

Figure S4. The maps of soil pH at 0-5 cm depth (A), 5-15 cm depth (B), 15-30 cm depth (C), 30-60 cm depth (D), 60-100 cm depth (E), and 100-200 cm depth (F) in Gansu Province.

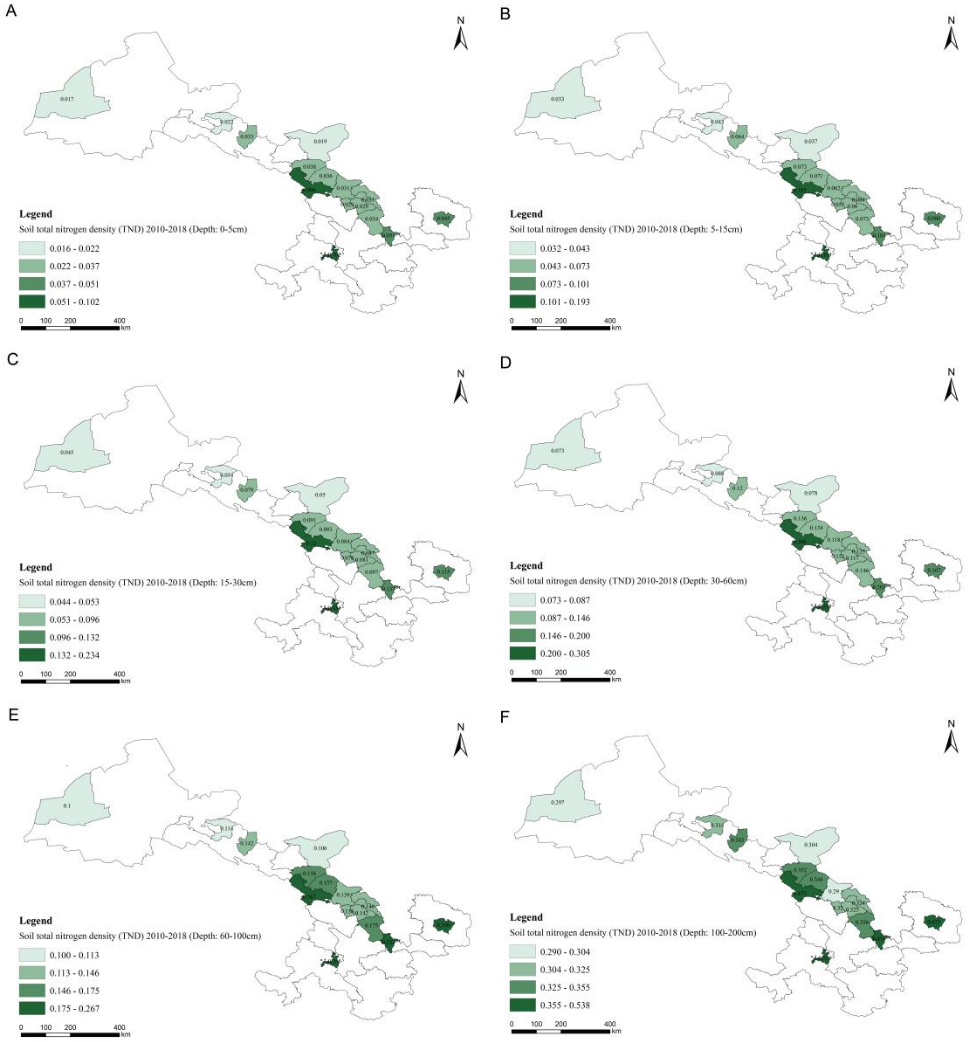

Figure S5. The maps of soil TND at 0–5 cm depth (A), 5-15 cm depth (B), 15-30 cm depth (C), 30-60 cm depth (D), 60-100 cm depth (E), and 100-200 cm depth (F) in Gansu Province. Abbreviations: TND, total nitrogen density.

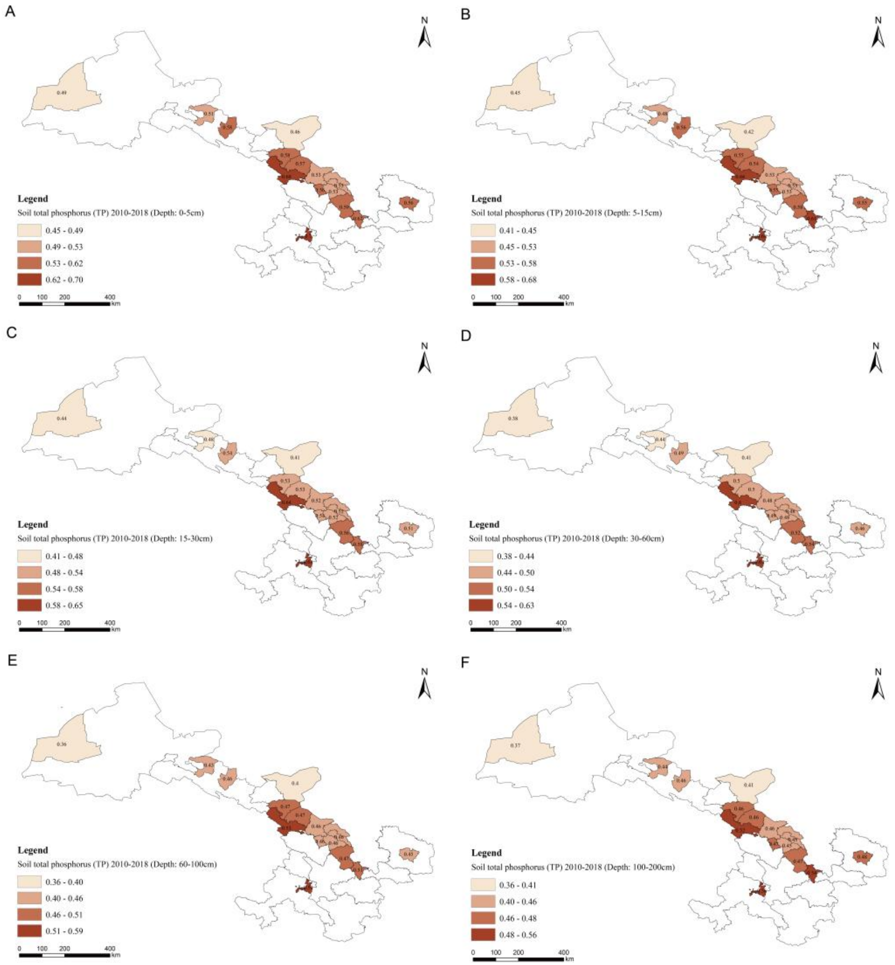

Figure S6. The maps of soil TP at 0-5 cm depth (A), 5-15 cm depth (B), 15-30 cm depth (C), 30-60 cm depth (D), 60-100 cm depth (E), and 100-200 cm depth (F) in Gansu Province. Abbreviations: TP, total phosphorus.

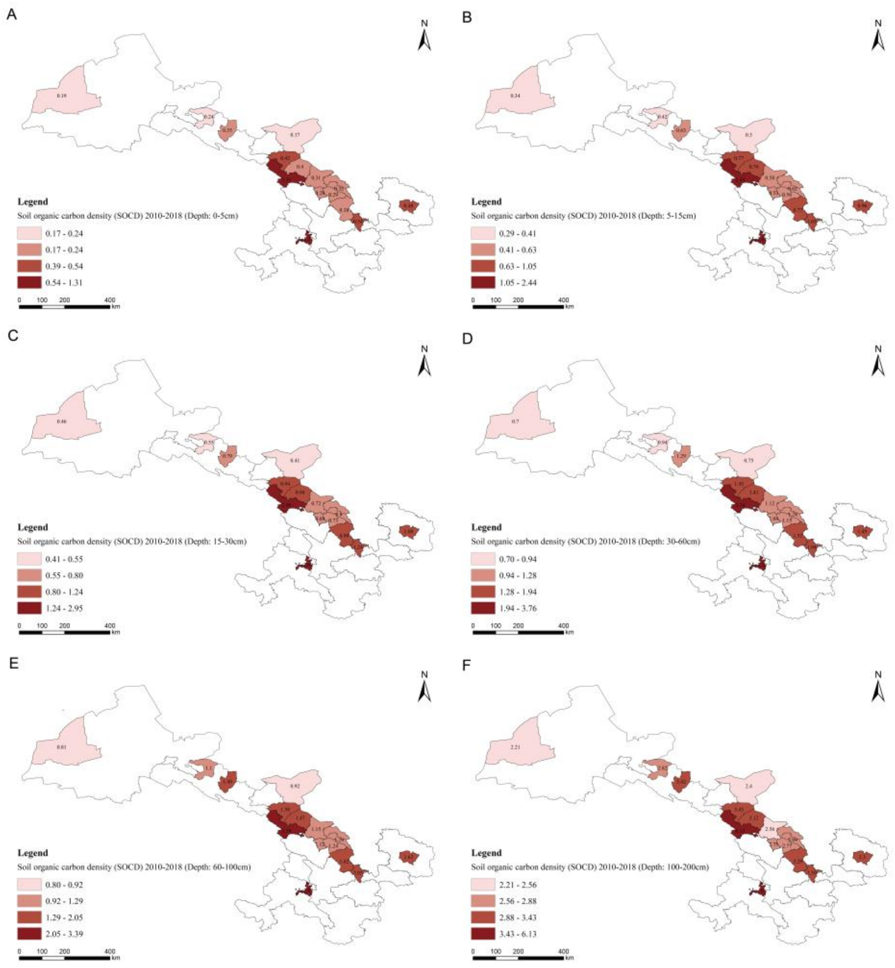

Figure S7. The maps of soil SOCD at 0-5 cm depth (A), 5-15 cm depth (B), 15-30 cm depth (C), 30-60 cm depth (D), 60-100 cm depth (E), and 100-200 cm depth (F) in Gansu Province. Abbreviations: SOCD, soil organic carbon density.

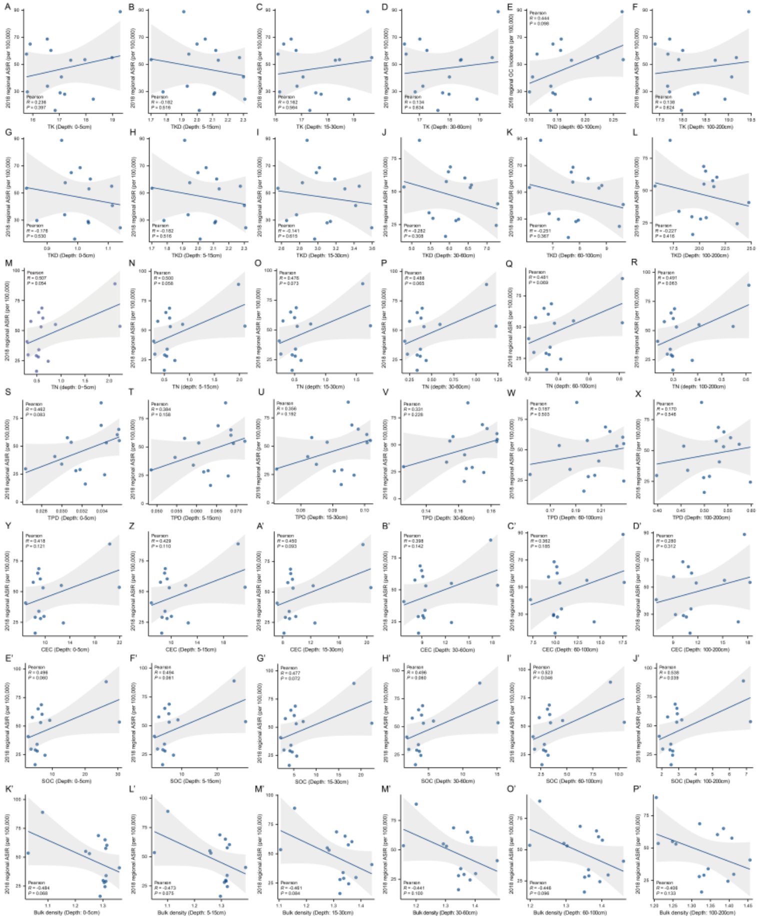

Figure S8. Scatter plots showing relationships between TK (A-F), TKD (G-L), TN (M-R), SPD (S-X), CEC (Y-D'), SOC(E'-J') and bulk density (K'-P') levels and ASIR (per 100,000 population) of gastric cancer in Gansu Province. Abbreviations: ASIR, age-standardized incidence rate; TK, total potassium; TKD, total potassium density; TN, total nitrogen; TPD: total phosphorus density; CEC, cation exchange capacity; SOC, soil organic carbon.

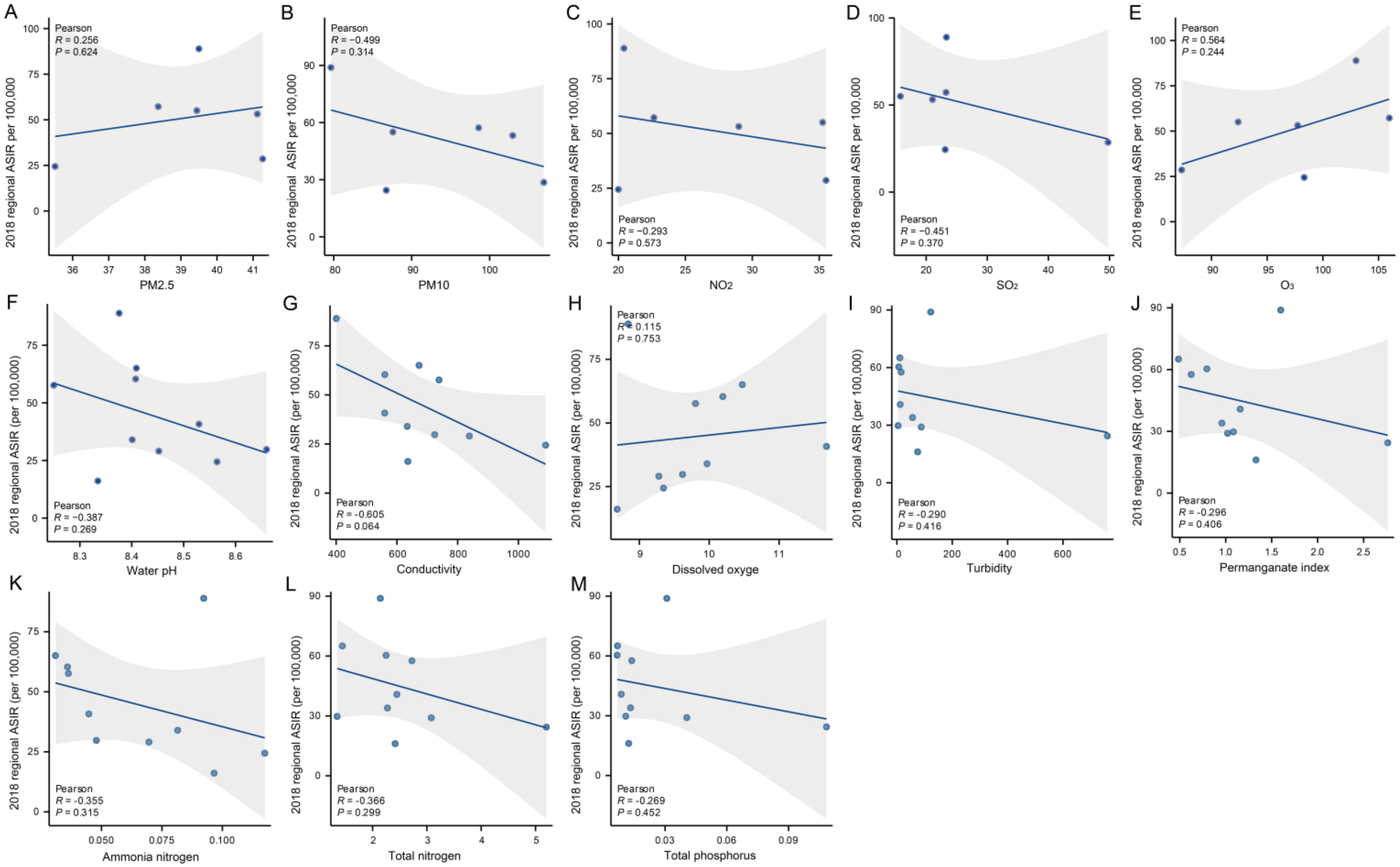

Figure S9. Scatter plots showing relationships between air (Panels A–E) and nature water parameters (Panels F–M) and ASIR (per 100 000 population) of gastric cancer in Gansu Province. ASIR – age-standardised incidence rate.

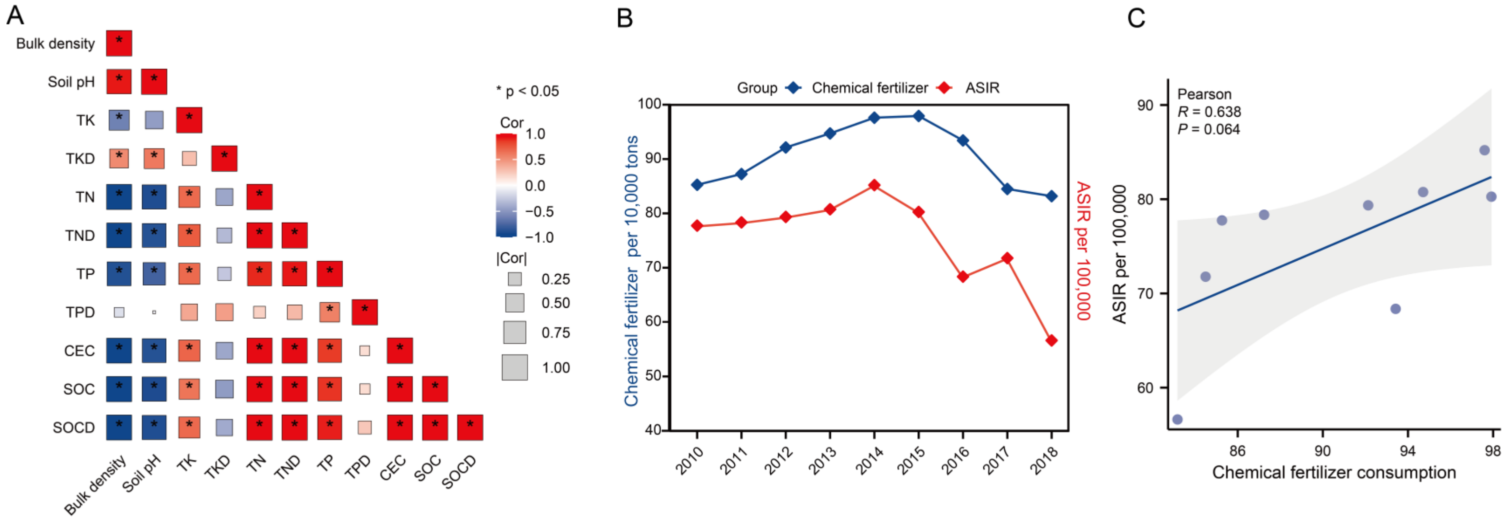

Figure S10. Panel A. Pearson's correlation coefficient across 11 soil indicators. The size of the square and colour depth are proportional to the absolute value of the correlation coefficient. Blue indicates a negative correlation, and red positive. Panel B. Annual trends of the applying quantity of chemical fertilizers and ASIR world of gastric cancer by year in Gansu Province, 2010–18. Panel C. Scatter plots showing relationships between the applying quantity of chemical fertilizers and ASIR (per 100 000 population) of gastric cancer in Gansu Province. *Statistically significant data. ASIR – age-standardised incidence rate, CEC – cation exchange capacity, SOC – soil organic carbon, SOCD – soil organic carbon density, TK – total potassium, TKD – total potassium density, TN – total nitrogen, TND –total nitrogen density, TP – total phosphorus, TPD – total phosphorus density.

Table S1. The ASIR and ASMR (China) per 100,000 population for gastric cancer in Gansu Province and total China.

Year	Number of incidence cases, all	Gansu			Number of incidence cases, all	China			IRR	95%CI	
		ASIR	95%CI			ASIR	95%CI				
2010	1633	71.05	67.61	74.50	45840	20.87	20.68	21.06	3.40	3.24	3.57
2011	1763	72.07	68.70	75.43	51820	23.57	23.37	23.77	3.06	2.91	3.20
2012	1655	71.16	67.73	74.59	66318	22.01	21.84	22.18	3.23	3.08	3.39
2013	1861	73.32	69.99	76.65	76243	21.60	21.45	21.75	3.39	3.24	3.55
2014	1921	77.09	73.65	80.54	90474	19.87	19.74	20.00	3.88	3.70	4.06
2015	1942	72.93	69.69	76.17	97948	18.73	18.61	18.85	3.89	3.72	4.07
2016	1660	62.04	59.05	65.02	111797	17.73	17.63	17.83	3.50	3.33	3.67
2017	1764	64.85	61.83	67.88	123200	16.78	16.69	16.87	3.86	3.68	4.05
2018	3133	42.60	41.11	44.10	141415	15.92	15.84	16.00	2.68	2.58	2.77
Year	Number of deaths, all	Gansu			Number of deaths, all	China			MRR	95%CI	
		ASMR	95%CI			ASMR	95%CI				
2010	1084	49.41	46.46	52.35	32958	14.30	14.15	14.45	3.45	3.25	3.66
2011	991	41.93	39.32	44.54	36840	16.19	16.02	16.36	2.59	2.43	2.75
2012	1091	49.69	46.75	52.64	47260	15.12	14.98	15.26	3.29	3.09	3.48
2013	1225	51.92	49.01	54.83	54613	14.91	14.78	15.04	3.48	3.28	3.68
2014	1191	49.14	46.35	51.93	64868	13.65	13.54	13.76	3.60	3.39	3.81

2015	1189	48.77	45.99	51.54	71864	13.19	13.09	13.29	3.70	3.49	3.91
2016	1267	48.32	45.66	50.98	82483	12.52	12.43	12.61	3.86	3.64	4.07
2017	1152	44.68	42.10	47.26	91166	11.95	11.87	12.03	3.74	3.52	3.96
2018	1996	26.90	25.72	28.08	103378	11.09	11.02	11.16	2.43	2.32	2.53

Abbreviations: ASIR, age-standardized incidence rate; ASMR, age-standardized mortality rate; IRR, incidence rate ratio; MRR, mortality rate ratio.

Table S2. The ASIR and ASMR per 100,000 population for gastric cancer in Gansu Province, 2010-2017.

Year	Number of incidence cases, all	ASIR									IRR	95%CI	
		Total	95%CI		Male	95%CI		Female	95%CI				
2010	1633	77.75	73.98	81.52	116.04	109.53	122.55	39.19	35.41	42.97	2.96	2.63	3.29
2011	1763	78.34	73.47	83.22	112.10	105.92	118.28	44.33	40.43	48.23	2.53	2.27	2.79
2012	1655	79.35	74.64	84.06	119.01	112.41	125.60	40.20	36.28	44.12	2.96	2.63	3.29
2013	1861	80.76	76.24	85.28	120.91	114.55	127.27	40.24	36.62	43.87	3.00	2.69	3.32
2014	1921	85.20	80.36	90.04	132.76	126.01	139.51	38.54	34.91	42.16	3.45	3.08	3.81
2015	1942	80.27	75.71	84.83	126.32	119.96	132.68	35.09	31.76	38.42	3.60	3.21	3.99
2016	1660	68.36	64.60	72.12	106.55	100.68	112.41	31.53	28.40	34.65	3.38	3.00	3.76
2017	1764	71.77	67.63	75.92	113.17	107.18	119.17	31.70	28.57	34.82	3.57	3.17	3.97
APC (2010-2014)		2.08			3.64			2.18					
APC (2014-2017)		-6.12			-5.40			-6.13					
AAPC		-1.52			-0.34			-3.83					

P value		>0.05			>0.05			<0.05					

Year	Number of deaths, all	ASMR									MRR	95%CI	
		Total	95%CI		Male	95%CI		Female	95%CI				
2010	1084	55.69	52.38	59.01	80.13	74.53	85.72	30.93	27.41	34.45	2.59	2.24	2.94
2011	991	46.66	43.75	49.56	72.44	67.34	77.55	20.11	17.44	22.79	3.60	3.06	4.14
2012	1091	57.06	53.68	60.45	84.14	78.34	89.93	30.34	26.79	33.89	2.77	2.40	3.15
2013	1225	57.72	54.49	60.96	86.22	80.61	91.82	29.21	25.99	32.43	2.95	2.57	3.33
2014	1191	54.54	51.44	57.64	85.69	80.17	91.20	24.04	21.13	26.94	3.56	3.08	4.05
2015	1189	57.09	53.84	60.33	84.68	79.05	90.31	30.39	27.06	33.72	2.79	2.43	3.14
2016	1267	54.29	51.30	57.28	84.96	79.62	90.31	24.82	21.99	27.65	3.42	2.98	3.87
2017	1152	51.06	48.11	54.01	78.72	73.49	83.95	24.64	21.76	27.51	3.20	2.77	3.62
APC (2010-2015)		1.66			3.29			0.41					
APC (2015-2017)		-5.34			-2.70			-6.97					
AAPC		-0.39			0.68			-1.75					
P value		>0.05			>0.05			>0.05					

Abbreviations: ASIR, age-standardized incidence rate; ASMR, age-standardized mortality rate; APC, annual percent change; AAPC, average APC; IRR, incidence rate ratio; MRR, mortality rate ratio.

Table S3. The average age of diagnosis cases and deaths of gastric cancer in Gansu Province during 2010 to 2018.

Year	Total		Male		Female	
	mean	SD	mean	SD	mean	SD
For diagnosis cases						
2010	60.19	10.60	60.43	10.37	59.51	11.23
2011	59.34	11.14	60.34	10.77	57.34	11.75
2012	61.48	10.77	61.65	10.03	60.94	12.80
2013	61.60	10.01	61.97	9.72	60.51	10.76
2014	63.19	10.31	63.45	9.91	62.30	11.58
2015	61.29	10.72	61.45	10.50	60.79	11.36
2016	62.11	10.54	62.53	10.19	60.75	11.50
2017	62.58	10.54	62.79	10.12	61.84	11.86
2018	63.89	10.79	64.17	10.11	63.11	12.49
For deaths						
2010	62.95	10.84	62.3	10.92	64.67	10.46
2011	62.48	10.75	63.13	10.38	60.19	11.72
2012	65.83	10.34	65.99	9.78	65.38	11.8
2013	63.75	12.76	64.88	11.66	60.42	15.02
2014	63.64	9.92	63.72	9.79	63.34	10.38

2015	66.22	11.16	66.35	10.90	65.85	11.68
2016	61.58	10.38	61.87	10.11	60.59	11.16
2017	65.65	10.07	65.65	9.71	65.66	11.12
2018	65.73	10.13	65.24	9.89	67.11	10.66

Table S4. The average age of diagnosis cases and deaths of gastric cancer in Gansu Province, 2018.

Region	Total		Male		Female	
	mean	SD	mean	SD	mean	SD
For diagnosis cases						
Lintan	61.15	11.07	60.36	9.82	63.30	13.92
Tianzhu	62.44	9.63	63.38	9.37	60.41	9.99
Qingcheng	62.52	12.37	61.87	13.11	63.83	10.9
Jingning	62.71	12.10	64.19	10.89	58.52	14.26
Gulang	63.06	9.98	62.84	9.19	63.74	12.2
Gaotai	63.20	9.06	63.58	8.81	61.42	10.24
Liangzhou	63.65	10.27	63.86	9.69	62.98	11.92
Jingtai	63.69	12.11	63.29	12.91	65.11	8.94
Jingyuan	64.24	11.63	65.68	10.18	61.42	13.71
Ganzhu	64.70	9.82	64.22	9.43	66.02	10.77
Huining	64.89	11.45	64.86	10.55	64.97	13.36

Pingchuan	65.2	12.49	66.49	10.72	61.11	16.66
Dunhuang	65.42	8.78	66.15	8.60	63.00	9.39
Minqin	65.61	11.17	66.37	10.10	62.97	14.14
Baiyin	67.62	10.89	68.09	10.71	66.42	11.47
			For deaths			
Lintan	62.54	10.26	61.77	9.06	65.20	13.69
Ganzhu	64.21	9.55	63.85	9.29	65.04	10.13
Gulang	65.22	9.25	64.46	8.76	67.67	10.41
Tianzhu	65.22	10.65	64.68	10.56	67.13	11.13
Jingning	65.54	11.13	65.02	10.44	67.27	13.20
Pingchuan	65.67	11.83	64.87	12.33	67.75	10.65
Liangzhou	65.70	9.66	65.06	9.66	67.33	9.50
Huining	66.07	10.96	64.74	10.7	69.70	10.94
Jingtai	66.63	11.39	66.53	11.12	66.95	12.54
Gaotai	66.67	7.21	66.93	6.97	65.44	8.57
Baiyin	66.87	10.59	66.45	10.44	67.89	11.52
Minqin	67.28	9.87	67.62	9.54	65.94	11.39
Dunhuang	67.34	8.75	68.34	8.22	62.50	10.41
Jingyuan	67.49	10.18	67.85	9.87	66.42	11.16
Qingcheng	69.27	10.12	69.93	10.32	67.67	9.87

Table S5. Soil information values of total China and Gansu Province.

Soil type	Gansu	China	Ratio
Soil pH (Depth: 0-5cm)	7.892	7.861	1.00
Soil pH (Depth: 5-15cm)	7.913	7.881	1.00
Soil pH (Depth: 15-30cm)	7.995	7.973	1.00
Soil pH (Depth: 30-60cm)	8.100	8.090	1.00
Soil pH (Depth: 60-100cm)	8.131	8.110	1.00
Soil pH (Depth: 100-200cm)	8.196	8.161	1.00
TN (Depth: 0-5cm)	1.156	0.996	1.16
TN (Depth: 5-15cm)	1.110	0.963	1.15
TN (Depth: 15-30cm)	0.900	0.785	1.15
TN (Depth: 30-60cm)	0.653	0.569	1.15
TN (Depth: 60-100cm)	0.521	0.454	1.15
TN (Depth: 100-200cm)	0.434	0.381	1.14
TND (Depth: 0-5cm)	0.060	0.049	1.22
TND (Depth: 5-15cm)	0.117	0.095	1.23
TND (Depth: 15-30cm)	0.143	0.117	1.23
TND (Depth: 30-60cm)	0.201	0.164	1.23

TND (Depth: 60-100cm)	0.210	0.174	1.21
TND (Depth: 100-200cm)	0.447	0.380	1.18
TP (Depth: 0-5cm)	0.613	0.580	1.06
TP (Depth: 5-15cm)	0.600	0.558	1.08
TP (Depth: 15-30cm)	0.566	0.536	1.06
TP (Depth: 30-60cm)	0.523	0.492	1.06
TP (Depth: 60-100cm)	0.496	0.464	1.07
TP (Depth: 100-200cm)	0.501	0.463	1.08
TPD (Depth: 0-5cm)	0.033	0.030	1.10
TPD (Depth: 5-15cm)	0.066	0.059	1.13
TPD (Depth: 15-30cm)	0.094	0.084	1.12
TPD (Depth: 30-60cm)	0.170	0.151	1.12
TPD (Depth: 60-100cm)	0.209	0.186	1.12
TPD (Depth: 100-200cm)	0.529	0.474	1.12
TK (Depth: 0-5cm)	18.176	17.411	1.04
TK (Depth: 5-15cm)	18.372	17.746	1.04
TK (Depth: 15-30cm)	18.566	17.760	1.05

TK (Depth: 30-60cm)	18.641	17.939	1.04
TK (Depth: 60-100cm)	18.740	17.891	1.05
TK (Depth: 100-200cm)	18.854	18.302	1.03
TKD (Depth: 0-5cm)	1.013	0.937	1.08
TKD (Depth: 5-15cm)	2.058	1.896	1.09
TKD (Depth: 15-30cm)	3.118	2.817	1.11
TKD (Depth: 30-60cm)	6.121	5.594	1.09
TKD (Depth: 60-100cm)	7.940	7.257	1.09
TKD (Depth: 100-200cm)	20.001	18.893	1.06
Bulk density (Depth: 0-5cm)	1.208	1.217	0.99
Bulk density (Depth: 5-15cm)	1.228	1.234	1.00
Bulk density (Depth: 15-30cm)	1.271	1.277	1.00
Bulk density (Depth: 30-60cm)	1.312	1.318	1.00
Bulk density (Depth: 60-100cm)	1.328	1.342	0.99
Bulk density (Depth: 100-200cm)	1.306	1.332	0.98
SOC (Depth: 0-5cm)	13.684	11.933	1.15
SOC (Depth: 5-15cm)	12.802	11.113	1.15

SOC (Depth: 15-30cm)	9.876	8.658	1.14
SOC (Depth: 30-60cm)	7.046	6.241	1.13
SOC (Depth: 60-100cm)	5.356	4.767	1.12
SOC (Depth: 100-200cm)	4.191	3.870	1.08
SOCD (Depth: 0-5cm)	0.702	0.581	1.21
SOCD (Depth: 5-15cm)	1.332	1.090	1.22
SOCD (Depth: 15-30cm)	1.551	1.276	1.22
SOCD (Depth: 30-60cm)	2.145	1.784	1.20
SOCD (Depth: 60-100cm)	2.123	1.785	1.19
SOCD (Depth: 100-200cm)	4.224	3.733	1.13

Abbreviations: TN, total nitrogen; TND, total nitrogen density; TP, total phosphorus content; TPD, total phosphorus density; TK, total potassium; TKD, total potassium density; CEC, cation exchange capacity; SOC, soil organic carbon; SOCD, soil organic carbon density.
